# Supplementary material for: Factors associated with mortality in children under five years old hospitalized for Severe Acute Malnutrition in Limpopo province, South Africa, 2014-2018: A cross-sectional analytic study
Source: PLoS One. 2020 May 8;15(5):e0232838. doi: 10.1371/journal.pone.0232838 (PMC7209205; doi:10.1371/journal.pone.0232838)
Supplement: S1 File — (PDF) [file pone.0232838.s001.pdf]

UNIVERSITEIT VAN PRETORIA
UNIVERSITY OF PRETORIA
YUNIBESITHI YA PRETORIA

Faculty of Health Sciences

The Research Ethics Committee, Faculty Health Sciences, University of Pretoria complies with ICH-GCP guidelines and has US Federal wide Assurance.
- FWA 00002567, Approved dd 22 May 2002 and Expires 03/20/2022.
- IRB 0000 2235 IORG0001762 Approved dd 22/04/2014 and Expires 03/14/2020.

**22 November 2018**

**Approval Certificate**
**New Application**

**Ethics Reference No: 551/2018**

**Title:** Risk factors associated with mortality in children under 5 years old with severe acute malnutrition in Limpopo Province, 2014-2018.

Dear Ms F Gavhi

The **New Application** as supported by documents received between 2018-11-15 and 2018-11-22 for your research,was approved by the Faculty of Health Sciences Research Ethics Committee on its quorate meeting of 2018-11-21.

Please note the following about your ethics approval:
- Ethics Approval is valid for 1 year and needs to be renewed annually by 2019-11-22.
- Please remember to use your protocol number (**551/2018**) on any documents or correspondence with the Research Ethics Committee regarding your research.
- Please note that the Research Ethics Committee may ask further questions, seek additional information, require further modification, or monitor the conduct of your research.

**Ethics approval is subject to the following:**
- The ethics approval is conditional on the research being conducted as stipulated by the details of all documents submitted to the Committee. In the event that a further need arises to change who the investigators are, the methods or any other aspect, such changes must be submitted as an Amendment for approval by the Committee.

- The REC granted a waiver for Informed Consent.

We wish you the best with your research.

**Yours sincerely**

**Dr R Sommers**; MBChB; MMed (Int); MPharMed,PhD
**Deputy Chairperson** of the Faculty of Health Sciences Research Ethics Committee, University of Pretoria

*The Faculty of Health Sciences Research Ethics Committee complies with the SA National Act 61 of 2003 as it pertains to health research and the United States Code of Federal Regulations Title 45 and 46. This committee abides by the ethical norms and principles for research, established by the Declaration of Helsinki, the South African Medical Research Council Guidelines as well as the Guidelines for Ethical Research: Principles Structures and Processes, Second Edition 2015 (Department of Health).*

Research Ethics Committee
Room 4-60, Level 4, Tswelopele Building
University of Pretoria, Private Bag X323
Arcadia 0007, South Africa
Tel +27 (0)12 356 3084
Email deepeka.behari@up.ac.za
www.up.ac.za

Fakulteit Gesondheidswetenskappe
Lefapha la Disaense tša Maphelo
